# Case report: Self-administration of alpha-1 antitrypsin therapy: a report of two cases

**DOI:** 10.3389/fphar.2023.1291677

**Published:** 2023-11-21

**Authors:** Ana M. Escribano Dueñas, Mónica Martín García, Begoña Tortajada Goitia, José Javier Arenas Villafranca

**Affiliations:** ^1^ Pulmonology Department, Hospital Universitario Costa del Sol, Marbella, Spain; ^2^ Day Hospital, Hospital Universitario Costa del Sol, Marbella, Spain; ^3^ Hospital Pharmacy Department, Hospital Universitario Costa del Sol, Marbella, Spain

**Keywords:** alpha-1 antitrypsin deficiency, disease burden, augmentation therapy, self-administration, independence, satisfaction

## Abstract

Intravenous augmentation therapy with human alpha-1 proteinase inhibitor for the management of respiratory disease is recommended for people with alpha-1 antitrypsin deficiency (AATD) who are nonsmokers or former smokers. Augmentation therapy usually requires weekly administration at the hospital or clinic and poses an additional burden for patients due to interference with daily life, including work and social activities. Self-administration is a useful alternative to overcome this limitation, but there is a lack of published information on clinical outcomes. We report two cases of individuals with AATD at different stages of the disease who were successfully managed with self-administered augmentation therapy, with increased satisfaction because of the independence gained, lack of interference with clinical stability, and no relevant safety issues.

## 1 Introduction

Alpha-1 antitrypsin deficiency (AATD) is a rare genetic disorder that poses a major clinical burden, mostly in terms of respiratory and liver disease, impacting quality of life and mortality (Miravitlles et al., 2022). Intravenous augmentation therapy with human alpha-1 proteinase inhibitor (A1PI) for the management of respiratory disease is recommended for people with AATD who are nonsmokers or former smokers (Sandhaus et al., 2016; Miravitlles et al., 2017). Augmentation therapy usually requires weekly administration at the hospital or clinic and poses an additional burden for patients due to interference with daily life, including work and social activities (Annunziata et al., 2021). Several strategies have been proposed to overcome this limitation: biweekly administration, home therapy and self-administration (Conde et al., 2023). The former is biochemically efficacious but lacks information on the impact on the long-term course of the disease. Home therapy and, especially, self-administration are useful alternatives and lead to increased independence of the patients. However, there is no information on clinical outcomes among patients who self-administer augmentation therapy.

We report two cases of individuals with AATD at different stages of the disease who were successfully managed with self-administered augmentation therapy.

## 2 Case description

### 2.1 Case 1

A 50-year-old woman who was employed full-time as a tourist guide, practiced sports daily and quit smoking 25 years ago was seen at our clinic complaining of dyspnea grade 1 on the Modified Medical Research Council (mMRC) scale. Spirometry showed a predicted forced expiratory volume in 1 s (FEV1) of 58.6%, a predicted forced vital capacity (FVC) of 86.3% and an FEV1/FVC ratio of 54%. The computed tomography scan revealed panacinar emphysema, and the patient was diagnosed with moderate chronic obstructive pulmonary disease (COPD) with exacerbations and no other comorbidities. Genetic testing showed genotype PI*ZZ and an A1PI serum concentration <20 mg/dl. The patient initiated treatment with a long-acting β2-agonist (LABA) twice daily and a long-acting muscarinic antagonist (LAMA) twice daily. To maintain patient independence, self-administration of a human alpha-1 proteinase inhibitor (Respreeza^®^ which, according to its labelling information, after first infusions that should be administered under the supervision of a healthcare professional, subsequent infusions can be administered by a caregiver or by the patient) was proposed and initiated.

Training on self-administration with the patient and her husband and daughter as back-ups was initiated at the day hospital by a nurse and included general knowledge on human A1PI, how to work under aseptic conditions, adequate reconstitution of the concentrate, performing the canalization, appropriate infusion of the drug, correct disposal of needles and other residuals, and how to handle complications that could arise during self-administration, including when to contact the day hospital. The patient and her family needed seven sessions of 45–60 min (including the administration of the drug). The main challenge reported by the patient was the fear of experiencing difficulties with canalization. Trough levels of A1PI were determined during the training sessions to adjust the dose of human A1PI, which was finally set at 5 g every 10 days, leading to A1PI levels >60 mg/dL. The patient was also included in an at-home support program for self-administration developed by the manufacturer of Respreeza^®^, which implies documenting each infusion and any associated incident/complication.

The patient has been in the self-administration program for 9 months without complaints of difficulties with the canalization and with no complications except for a single event of blood reflux by the catheter that was solved with the help of the clinic by phone. In the last visit, the patient had dyspnea grade 0 on the mMRC scale, a predicted FEV1 of 65% and had not experienced exacerbations; serum levels of A1PI at 6 and 9 months were 64 and 68 mg/dL, respectively. Currently, the patient reports regular physical activity, full-time work and long-distance travel without limitations.

### 2.2 Case 2

A 68-year-old currently retired male engineer who had quit smoking 30 years ago presented to the hospital. Twelve years ago, he showed dyspnea grade 1 on the mMRC scale and spirometry showed a predicted FEV1 of 72%, a predicted FVC of 118% and an FEV1/FVC ratio of 48%; CT scan revealed panacinar emphysema, and the patient was diagnosed with mild COPD. The patient also showed dyslipidemia and benign prostate hyperplasia. The genotype was PI*ZZ, and the serum level of A1PI was 20–30 mg/dL. Three years after diagnosis, lung function worsened, showing a predicted FEV1 of 60%, a predicted FVC of 70% and an FEV1/FVC ratio of 63%. The patient initiated augmentation therapy with a human alpha-1 proteinase inhibitor (Prolastin^®^, which, according to its labelling information, should be administered by a healthcare professional) 17 gr biweekly at the day hospital. Augmentation therapy interfered greatly with his working activity, and the patient showed several determinations of A1PI serum concentration below 50 mg/dL.

In February 2020, during the COVID-19 lockdown, due to fear of being infected in the hospital, the patient discontinued augmentation therapy and maintained treatment with a LABA and LAMA. Six months later, the patient visited the clinic with severe hypoxemia and deterioration of lung function with a predicted FEV1 of 52%. Due to the patient’s concerns about the risk of COVID-19 infection, self-administration was proposed with Respreeza^®^ 5 gr weekly. His wife would be responsible for the administration at home and was trained by a nurse with an identical program as in Case 1 but needed 6 sessions. His wife reported no concerns or challenges during the training sessions.

The patient has been on self-administered therapy for 32 months, presenting only one mild COPD exacerbation. Currently, the patient shows dyspnea grade 2 on the MRC scale, a predicted FEV1 of 55% and an oxygen saturation level of 95%. The patient continues treatment with a LABA and LAMA and Respreeza^®^ 5 gr weekly, and his A1PI serum level is >59 mg/dL. He has reported no incidents or adverse events during self-administration and reports that self-administration allows him to travel for his social activities.

## 3 Discussion

Self-administration of intravenous drugs has been successfully performed in patients who require intravenous antibiotics (Tonna et al., 2019), patients with hemophilia (Schrijvers et al., 2012; Khair et al., 2015) and those with angioedema (Bygum et al., 2009; Craig, 2013; Zanichelli et al., 2018). The limited available patient-reported information on home-based administration and self-administration of augmentation therapy among patients with AATD indicates that clinic-based administration of augmentation therapy interferes greatly with patients’ lives (Annunziata et al., 2021) and that the greatest perceived benefit of self-administration is increased independence (Sandhaus and Boyd, 2018). Our results in two different scenarios, initiating self-administered augmentation therapy and switching from clinic-based to self-administration of augmentation therapy, are consistent with that major benefit of self-administration therapy. Although this has not been studied among patients with AATD, experience with self-administration of immunoglobulin G therapy (Ritchie et al., 2022) suggests that self-administration among individuals with AATD could be associated with reduced costs from the healthcare payer perspective.

The selection of patients and their training are key for the success of self-administration of augmentation therapy (Herth et al., 2021), with the latter from the patient’s perspective being the critical factor for adopting this treatment modality (Colello et al., 2022). For completely different reasons, in these two cases, self-administration was adapted to the patients’ needs: independence for an active life in one case and fear of being infected with COVID-19 during a hospital visit in the other; both cases share the patients’ need for avoiding clinic-based administration of augmentation therapy. The selection of these patients is consistent with a recent experts’ recommendations for the implementation of the self-administration of augmentation therapy that establish the following objectives: to empower the patient to actively manage and control the disease, to promote family/work conciliation and patient independence, to avoid nosocomial respiratory infections in the context of the COVID-19 pandemic, and/or to reduce the costs associated with augmentation therapy (Torres-Durán et al., 2023). Both cases needed 6-7 training sessions of 45 min, which is double the 2-3 sessions on average reported in a study in the United States (Herth et al., 2021). Differences between the Spanish and US health systems could explain this discrepancy. However, our experience is consistent with that reported for self-administration among individuals with hemophilia in the Netherlands (Schrijvers et al., 2012).

Overall, these two cases suggest that self-administration of augmentation therapy in patients with AATD is an appropriate option for some patients, is associated with increased satisfaction because of the independence gained, and does not appear to interfere with clinical stability or be associated with relevant safety issues.

## Data Availability

The raw data supporting the conclusion of this article will be made available by the authors, without undue reservation.

## Abbreviations

AATD, Alpha-1 antitrypsin deficiency; A1PI, alpha-1 proteinase inhibitor; COPD, chronic obstructive pulmonary disease; FEV1, forced expiratory volume in 1 s; FVC, forced vital capacity; LABA, long-acting β2-agonist; LAMA, long-acting muscarinic antagonist; MRC, Medical Research Council.

## References

[B1] Annunziata A. Lanza M. Coppola A. Andreozzi P. Spinelli S. Fiorentino G. (2021). Alpha-1 antitrypsin deficiency: home therapy. Front. Pharmacol. 12, 575402. 10.3389/fphar.2021.575402 33935692 PMC8082418

[B2] Bygum A. Andersen K. E. Mikkelsen C. S. (2009). Self-administration of intravenous C1-inhibitor therapy for hereditary angioedema and associated quality of life benefits. Eur. J. Dermatol. 19, 147–151. 10.1684/ejd.2008.0603 19264579

[B3] Colello J. Ptasinski A. Zhan X. Kaur S. Craig T. (2022). Assessment of patient perspectives and barriers to self-infusion of augmentation therapy for alpha-1 antitrypsin deficiency during the COVID-19 pandemic. Pulm. Ther. 8, 95–103. 10.1007/s41030-022-00182-z 35067906 PMC8784277

[B4] Conde B. Costa F. Gomes J. Lopes A. P. Mineiro M. A. Rodrigues O. (2023). Expert perspectives on the management of alpha 1-antitrypsin deficiency. Acta Med. Port. 36, 49–54. 10.20344/amp.18497 35848753

[B5] Craig T. J. (2013). Recent advances in hereditary angioedema self-administration treatment: summary of an international hereditary angioedema expert meeting. Int. Arch. Allergy Immunol. 161, 26–27. 10.1159/000351241 23689242

[B6] Herth F. J. F. Sandhaus R. A. Turner A. M. Sucena M. Welte T. Greulich T. (2021). Alpha 1 antitrypsin therapy in patients with alpha 1 antitrypsin deficiency: perspectives from a registry study and practical considerations for self-administration during the COVID-19 pandemic. Int. J. Chron. Obstruct. Pulmon. Dis. 16, 2983–2996. 10.2147/copd.S325211 34754184 PMC8570922

[B7] Khair K. Meerabeau L. Gibson F. (2015). Self-management and skills acquisition in boys with haemophilia. Health Expect. 18, 1105–1113. 10.1111/hex.12083 23711015 PMC5060849

[B8] Miravitlles M. Dirksen A. Ferrarotti I. Koblizek V. Lange P. Mahadeva R. (2017). European respiratory society statement: diagnosis and treatment of pulmonary disease in α_1_-antitrypsin deficiency. Eur. Respir. J. 50, 1700610. 10.1183/13993003.00610-2017 29191952

[B9] Miravitlles M. Herepath M. Priyendu A. Sharma S. Vilchez T. Vit O. (2022). Disease burden associated with alpha-1 antitrypsin deficiency: systematic and structured literature reviews. Eur. Respir. Rev. 31, 210262. 10.1183/16000617.0262-2021 35321931 PMC9488933

[B10] Ritchie B. Martins K. J. B. Tran D. T. Blain H. Richer L. Klarenbach S. W. (2022). Economic impact of self-administered subcutaneous versus clinic-administered intravenous immunoglobulin G therapy in Alberta, Canada: a population-based cohort study. Allergy Asthma Clin. Immunol. 18, 99. 10.1186/s13223-022-00735-6 36434668 PMC9700869

[B11] Sandhaus R. A. Boyd B. S. (2018). “Alpha 1 antitrypsin therapy: a satisfaction survey of individuals self-administering,” in A46. Topics in alpha-1-antitrypsin deficiency (San Diego, CA: American Thoracic Society), A1758.

[B12] Sandhaus R. A. Turino G. Brantly M. L. Campos M. Cross C. E. Goodman K. (2016). The diagnosis and management of alpha-1 antitrypsin deficiency in the adult. Chronic Obstr. Pulm. Dis. 3, 668–682. 10.15326/jcopdf.3.3.2015.0182 28848891 PMC5556762

[B13] Schrijvers L. H. Beijlevelt-Van der Zande M. Peters M. Schuurmans M. J. Fischer K. (2012). Learning intravenous infusion in haemophilia: experience from The Netherlands. Haemophilia 18, 516–520. 10.1111/j.1365-2516.2012.02752.x 22292416

[B14] Tonna A. Anthony G. Tonna I. Paudyal V. Forbes-McKay K. Laing R. (2019). Home self-administration of intravenous antibiotics as part of an outpatient parenteral antibiotic therapy service: a qualitative study of the perspectives of patients who do not self-administer. BMJ Open 9, e027475. 10.1136/bmjopen-2018-027475 PMC6347967 30782762

[B15] Torres-Durán M. López-Campos J. L. Calle Rubio M. Montero-Martínez C. Priegue Carrera A. Amaro Rodríguez R. (2023). Recommendations for the implementation of the self-administration of alpha-1 antitrypsin. Int. J. Chron. Obstruct Pulmon Dis. 18, 1691–1700. 10.2147/copd.S410611 37559832 PMC10408674

[B16] Zanichelli A. Azin G. M. Cristina F. Vacchini R. Caballero T. (2018). Safety, effectiveness, and impact on quality of life of self-administration with plasma-derived nanofiltered C1 inhibitor (Berinert®) in patients with hereditary angioedema: the SABHA study. Orphanet J. Rare Dis. 13, 51. 10.1186/s13023-018-0797-3 29631595 PMC5891972

